# Supplementary material for: Willingness to receive COVID-19 vaccine and associated factors among adult chronic patients. A cross-sectional study in Northwest Ethiopia
Source: PLoS One. 2022 Jul 12;17(7):e0269942. doi: 10.1371/journal.pone.0269942 (PMC9275707; doi:10.1371/journal.pone.0269942)
Supplement: Attachment — Submitted filename: Response letter to reviewers.docx [file pone.0269942.s002.docx]

**Point-by-point response letter**

**Title: Willingness to receive COVID-19 vaccine and associated factors among adult chronic patients. A cross-sectional study in Northwest Ethiopia**

**Dear, Editors of PLOS ONE**

I want to convey our gratitude for reviewing our research on behalf of all authors.We appreciate your time and effort and that of the associate editors and reviewers in offering feedback on our paper. We value your and the reviewers' thoughtful comments and significant improvements to our manuscript. We tried to address the editor's and reviewers' issues and suggestions. The following is a detailed response to the reviewer's suggestions and comments.

Finally, we certify that this paper has not been previously published and is not currently being considered by another publication. All authors have approved the manuscript and agreed to resubmit it to PLOS ONE.

**With kind regards!**

On behalf of the co-authors

Masresha Derese Tegegne, [MPH in Health informatics]

**Response to editor’s**

We have edited and amended the manuscript based on your suggestions and comments. We appreciate your constructive criticism and suggestions for enhancing this work. Please find a detailed response to the editor's analysis and recommendations below.

**Editor comments**

**When submitting your revision, we need you to address these additional requirements.**

1. Please ensure that your manuscript meets PLOS ONE's style requirements, including those for file naming. The PLOS ONE style templates can be found at

<https://journals.plos.org/plosone/s/file?id=wjVg/PLOSOne_formatting_sample_main_body.pdf>  and<https://journals.plos.org/plosone/s/file?id=ba62/PLOSOne_formatting_sample_title_authors_affiliations.pdf>

**Replay#1:** Thanks for the notifications; we confirm that our manuscript meets the PLoS one style requirements.

2. Please provide additional details regarding participant consent. In the ethics statement in the Methods and online submission information, please ensure that you have specified what type you obtained (for instance, written or verbal, and if verbal, how it was documented and witnessed). If your study included minors, state whether you obtained consent from parents or guardians. If the need for consent was waived by the ethics committee, please include this information.

**Replay#2:** Thank you for your constructive suggestions. As we explained in the Ethics approval and consent to participate section, each patient signed a consent form after being informed about the study's purpose. This is stated explicitly in the manuscript and submission form.

3. In your Data Availability statement, you have not specified where the minimal data set underlying the results described in your manuscript can be found. PLOS defines a study's minimal data set as the underlying data used to reach the conclusions drawn in the manuscript and any additional data required to replicate the reported study findings in their entirety. All PLOS journals require that the minimal data set be made fully available. For more information about our data policy, please see <http://journals.plos.org/plosone/s/data-availability>.

**Replay#3:** Thank you for informing us. All of the data created or analyzed during this investigation is contained in this version of the paper and the supplementary file.

**Response to reviewer(s') comments**

On behalf of all authors, I'd like to thank the reviewers for their constructive suggestions, which helped to improve the paper's scientific foundation. The criticism was well-received, and most of the reviewers' suggestions were enhanced. Throughout the document in the tracked change file, certain modifications are highlighted. Please see the blue text below for a point-by-point response to the reviewers' complaints and proposals.

**Reviewer #1 Evaluation**

**General Comments**

*“*I was invited to review the paper entitled:” Willingness to receive COVID-19 vaccine and associated factors among adult chronic patients. A cross-sectional study in Northwest Ethiopia”. The study aimed to investigate willingness to receive COVID-19 vaccination and factors affected it among adult with chronic diseases. There has been a great deal of research in this area internationally, and I did not notice any novelty in the results of this study from an international viewpoint*”.*

**Replay:** Dear reviewer, thank you so much for taking the time to review our work!

Even though there are other international studies on the topic, this one is unique in that it concentrates on chronic patients. Chronic patients were more likely than other population groups to get COVID-19 infection. As a result, knowing their willingness will provide important data for vaccine camping in the research area. Aside from that, there is little evidence conducted on chronic patients in the study area. In general, knowing chronic patients' desire to receive the COVID-19 vaccine and associated factors is the best evidence at this moment.

***“*There are also several concerns with this study*”.***

1. Throughout the Background section, there are many inappropriate citations. For example, the authors cite reference number [9] for the history of vaccine development. However, this is a cross-sectional study to clarify vaccine hesitancy in Spain and is not an appropriate citation. The same thing happened with reference numbers [10], [11]. Reference number [16] is also inappropriate for citation because it is about vaccine hesitancy before COVID-19. The author should research previously research, and provide appropriate citations.

**Reply#1**. Thank you for the valid comments. This will significantly improve the quality of our research. We made significant changes to all of the referenced sources, and we certify that we cited the original reference that reported the notion in this version of the manuscript. Please see the first page of the introduction, lines number, 11,14,17,30 and the second page of the introduction, lines number 2 for your recommendations.

1. The sampling method of the target participants is very unclear.

**For example**

1.1. What exactly is a chronic disease?

**Reply#1.1:** Thank you for your feedback; in this revised manuscript, we have explained that “Patients with chronic diseases such as chronic liver disease, chronic kidney disease, hypertension, diabetes mellitus, and heart failure were included in this study”. Please have a look at the Study populations and eligibility criteria section on lines number 7, and 8 for further information.

1.2. Who diagnosed the chronic diseases and how?

**Reply#1.2**: Thank you for your informative feedback: Chronic disease was diagnosed using a set of clinical features, laboratory findings, and imaging results. Furthermore, when serum IgG levels are relatively high, it is referred to as chronic illness. The treating physicians determined the diagnosis based on the criteria given above. This study comprised patients who had their follow-up at the University of Gondar specialized hospital outpatient department. Please see the details in the study populations and eligibility criteria section on lines number 8,9 and 10.

1.3. Who made the decision to exclude the patients?

**Reply#1.3:** Thank you for your valuable input; it significantly improves the quality of our paper. Before data collection, one day of training was given to the two supervisors and eight data collectors who were involved in the data collection procedure. As a result, supervisors and data collectors under the strict supervision of the investigator made the decision to omit the participants based on the information supplied in the inclusion and exclusion criteria. Please see line number 9-11 for the data collection tool and quality control section.

1.4. What was the total number of patients who met the inclusion and exclusion criteria?

**Reply#1.4:** Thanks for your comments: As highlighted in study design and setting section on line number 30 the university of Gondar’s specialized hospital outpatient department serves approximately 6000 chronically ill patients who are followed up on a regular basis based on their unique diseases and severity of illness.

1.5. How were the participants randomly selected?

These points are not described in the current manuscript, and reproducibility is poor. At least in the current description, it cannot be called “random sampling technique”. Moreover, I am concerned about the possibility of a large selection bias in this paper.

**Reply#1.5** Thank you for your feedback: Your suggestion is helpful:

To select study participants, a systematic random sampling process was applied. Every day, an average of 150 chronic patients visit the outpatient departments of the University of Gondar specialized Hospital. A total of 2,250 chronic patients were predicted during the data collection period (15 working days). By dividing the total population by sample size (423), the sample interval was 5. In the first interval, the lottery method was used, and in the subsequent intervals, every fifth chronic patient was chosen until we had a total of 423 samples. Please have a look at the sampling procedure section.

2. The validity and reliability of the questionnaire (or survey item) is unknown.
   The authors described the translation procedure of the questionnaire, but there is no description of the validity and reliability of the original questionnaire.
   Also, the translation procedure is not sufficiently described. (Reference: <https://pubmed.ncbi.nlm.nih.gov/15804318/>)

**Reply#2:** Thank you for the valid comments, we would like to thank the reviewers for providing the right evidence on the translational procedure. As highlighted in the data collection and quality control section on lines number 13-20, the reliability and validity of the all-composite variables used in the study were determined by using the Cronbach alpha results from the pre-test. The Cronbach alpha scores on COVID-19 vaccine knowledge (Cronbach alpha=0.78), COVID-19 vaccine attitudes (Cronbach alpha=0.92), perceived susceptibility to COVID-19 (Cronbach alpha=0.77), perceived COVID-19 vaccination benefit (Cronbach alpha=0.75) and perceived potential vaccine harms (Cronbach alpha=0.76) were on the acceptable range. Besides this, as indicated in the data collection tool and quality control section on lines number 12,13, best practice was considered by evaluating the pre-test results and by describing the translation procedure of the questionnaire.

3. Looking at Table 1, chronic diseases do not include important chronic diseases, such as cancer and COPD. (Reference: <https://www.cdc.gov/coronavirus/2019-ncov/science/science-briefs/underlying-evidence-table.html>). I believe the point of this study is that it targeted patients with chronic diseases. Therefore, what is defined as chronic disease and how it is assessed are important points of this study. These should be clearly stated in the Methods section and the Results section.

**Reply#3:** We found your comments extremely helpful, and thanks for your comments. We didn't forget about the important chronic patients you mentioned; this is a writing issue that we addressed in Table 1 under Chronic illness [other categories]. Patients with psychiatric, arthritis, cancer, interstitial lung disease, chronic obstructive pulmonary disease, neurodegenerative disorders like Alzheimer's and Parkinson's, and inflammatory bowel diseases were studied. For more information, please see the footnote in Table 1.

4. The author described that a self-administered questionnaire was used in abstract and methods section. However, the authors state in the Limitation section that they conducted an Interview, which is inconsistent. Improvement of these above points is essential to consider the quality and importance of this paper. The reviewer reserves other comments at this moment.

**Reply#4:** Thanks for the valid insights: We confirmed that this was a typing mistake, and the authors performed face-to-face interviews with study participants who were eligible using a validated data collection tool. Please see line number 8 in the data collection tool and quality control section, as well as the abstract section.

**Reviewer #2 Evaluation**

Authors can consider the following suggestions:

1. Should update information. For instance, the total number of infected patients and death tolls caused by the pandemic. The authors have used old data in the introduction.

**Reply#1.** Thank you for your feedback. This is crucial to the study's credibility. We used the current prevalence of COVID-19 cases and deaths in accordance with your ideas. Please review the first parageraph on introductions.

2. Rationale for conducting this study on ‘patients suffering chronic diseases’ should be justified with the help of statistics and existing literature.

**Reply#2.** I appreciate your suggestion. This will be extremely beneficial to the quality of our research. We attempted to justify the significance of the study on chronic patients, as mentioned starting from line number 27, on the first page of the introductory section by using existing evidence. “*Even while everyone benefits from the vaccination, not everyone benefits equally. Those at a higher risk of contracting COVID-19 infection, more likely to get very unwell once infected, or at a higher risk of developing complications and dying from the disease will benefit the most from vaccination [**18**,* *19**]. Patients with chronic conditions such as diabetes, hypertension, cardiovascular, renal, and cerebrovascular diseases were given priority for vaccination since they were more likely to be affected by this virus and have a significant clinical burden from COVID-19 [**18**,* *20**].* ”. Aside from that, the importance of this study was stated on the second page of the introductory section, specifically lines 12-19.

3. It seems, the study was conducted before the planned vaccination campaign (end of 2021). That said, readers do not know what happened when the vaccination campaign actually started! Thus, the authors should be informed about the updated situation with reference to other parts of the world.

**Reply#3.** Thank you for your remarks, which we found incredibly helpful. We attempted to articulate the vaccination campaign in many countries throughout the world, as highlighted on the second page of the introduction section on lines 3-11.

4. It is good that the authors have explained the inclusion criteria, but it is not clear if they included any minor participants (aged less than 18 years). Also, the exclusion criteria require justification – why those groups of patients were excluded.

**Reply#4.** We found your comments extremely helpful, and thanks for your comments. In the study population and eligibility criteria section, we explained our inclusion criteria in detail on lines 7-12. Aside from this, as the title suggests, the research focuses on adult chronic patients. As a result, in the Ethiopian context, adult refers to a group of persons over the age of 18, hence the study automatically excludes those under the age of 18. Furthermore, we excluded patients who were mentally ill, unable to talk, or who had a significant hearing loss since they were unable to respond to questions about their willingness and permission. Please review the study population and eligibility criteria section, lines 12-15.

5. The manuscript should be thoroughly checked for grammatical accuracy.

**Reply#5.** Thank you very much for forwarding your comments. We certified that we attempted to improve the writing in this version of the manuscript. Please check over the entire document in the tracked change file; the work was reviewed for improved presentation by the writers and experienced English language specialists.

6. The study shows that over 54% of participants were willing to receive the vaccine. Well, did the participants mention and/or do the authors have any information on why they were not willing to receive the vaccine? It would be good to know why they were not willing to receive the vaccine.

**Reply#6.** Thank you for the comment! In addition to giving descriptive findings, this study focuses on identifying possible factors and their strength of association with the willingness. The descriptive findings and their regression associations of identified factors in prior investigations were taken into account. As a result, we examined a variety of factors, including perceived possible vaccine harm, lack of knowledge, and a negative attitude toward the COVID-19 vaccine. Please have a look at the result section.

7. The authors mentioned the proportion of the respondents willing to receive the vaccine. But were they informed about their comorbidities and possible side effects of receiving the vaccine?

**Reply#7.** Thank you for taking the time to share your thoughts. During the research period, the Ethiopian public health institute and the ministry of health enhanced their awareness. The Ethiopian Public Health Institute sends out two short messages about COVID-19, comorbidities, and possible adverse effects, as well as a helpline from the Ministry of Health.
