# Supplementary material for: Willingness to receive COVID-19 vaccine and associated factors among adult chronic patients. A cross-sectional study in Northwest Ethiopia
Source: PLoS One. 2022 Jul 12;17(7):e0269942. doi: 10.1371/journal.pone.0269942 (PMC9275707; doi:10.1371/journal.pone.0269942)
Supplement: Attachment — Submitted filename: Response letter to reviewers.docx [file pone.0269942.s003.docx]

**Point-by-point response letter**

**Title: Willingness to receive COVID-19 vaccine and associated factors among adult chronic patients. A cross-sectional study in Northwest Ethiopia**

**Dear, Editors of PLOS ONE**

I want to express our gratitude on behalf of all of the authors for taking the time to read our paper, "Willingness to receive COVID-19 vaccine and associated factors among adult chronic patients. A cross-sectional study in Northwest Ethiopia ".

We appreciate your time and work in providing input on our manuscript for the second time and those of the associate editors and reviewers. We appreciate your careful remarks and significant improvements to our work and those of the reviewers. We attempted to resolve the issues and ideas raised by the editor and reviewers. The following is a comprehensive response to the reviewer's opinions and criticisms.

Finally, we certify that this paper has not been previously published and is not currently being considered by another publication. All authors have approved the manuscript, and they have agreed to resubmit it to PLOS ONE Journal.

**With kind regards!**

On behalf of the co-authors

Masresha Derese Tegegne, [MPH in Health informatics]

**Response to editor’s**

We appreciate your constructive criticism and suggestions for enhancing this work. We have edited and amended the manuscript based on your suggestions and comments. Please find a detailed response to the editor's analysis and recommendations below.

**Editor comments**

1. Please review your reference list to ensure that it is complete and correct. If you have cited papers that have been retracted, please include the rationale for doing so in the manuscript text, or remove these references and replace them with relevant current references. Any changes to the reference list should be mentioned in the rebuttal letter that accompanies your revised manuscript. If you need to cite a retracted article, indicate the article’s retracted status in the References list and also include a citation and full reference for the retraction notice.

**Replay#1:** Thank you for the notices; we certify that we critically checked our references and that no references have been retracted.

2. English language correction is deemed necessary

**Replay#2:** Thank you very much for forwarding your comments. We certified that we attempted to improve the writing in this version of the manuscript. Please check over the entire document in the tracked change file; the work was reviewed for improved presentation by the writers and experienced English language specialists.

**Response to reviewer(s') comments**

Dear Reviewer#1: We appreciate your valuable suggestions for the second time, which are crucial in refining the paper's scientific foundation. Most of your requests were improved as a result of the feedback. In the tracked change file, some changes are highlighted throughout the document. Please see the complete responses below for a thorough response to your complaints and recommendations.

**Reviewer #1 Evaluation**

**General Comments**

 “I confirm that the revision has greatly improved this manuscript.”

**Replay#:** Thank you for your kind words; your suggestions were valuable, and we will use them to improve the scientific foundations of our research.

“However, there are still several points that need to be improved”.

**Background section**
1. The authors cited Wikipedia as the source for the description of COVAX (Reference number: 10), but I think that's not adequate. I believe that the information on Wikipedia is inaccurate because an unspecified number of users can freely change the description. Please consider citing a official website, such as WHO website, or article about the COVAX description.

**Replay#1:** We appreciate your comments; your concerns have been considered, and we have cited the official websites indicated on the WHO web pages. Please see line 14 in the introduction section for more information.

**Methods section**
2. Please describe the pre-test in more detail. Who was the target participant, and how many were eligible, etc.

**Replay#2:** Thanks for your suggestions, as it is highlighted in the data collection tool and quality control section; *"A pre-test involving 5% of the study population was conducted outside of the study area, at the Gondar town health center (Poli health center). Patients with chronic diseases in the outpatient department took part in the pre-test".* Please see the data collection and quality control section on line number 11-13 for further details.

3. The authors cited recommendations by the ISPOR task force in their description about the pre-test (Reference number: 40). But it is not clear what exactly they did. For example, did the authors modify the translated text based on the pre-test results, or did they just do cognitive debriefing and proofreading? Please describe in more detail what the authors did specifically based on the results of the pre-test.

**Replay#3:** Thank you for your useful feedback; “*Based on the pre-test results, we made as few adjustments as possible to data collection instruments before the actual data collection”*. Please see the data collection and quality control section on line number 14.

4. I understood that the authors evaluated all indicators using interview method. However, I think the current description in methods and abstract section do not clearly state that they have been evaluated in interviews. In addition, there is still the description " questionnaire" in part of the methods section, which is confusing. Please make these points clearer.

**Replay#4:** Thank you for your well-considered remarks. "Data was collected from eligible study participants using a face-to-face interview technique in the local language (Amharic)." Please look at lines 9 and 8, respectively, for the abstract and method section.

**Result section**
5. For the 22 excluded persons out of 423, please describe the reasons for their exclusion in the results section. How many refused to cooperate with the study? How many met the exclusion criteria such as hearing impairment?

**Replay#5:** Thank you for your feedback; As highlighted in lines number 15-17 in the result section, 19 people were judged ineligible for the study because they met the exclusion criteria, and 3 people were refused participation.

**Reference**
6. I think some of the descriptions in the Reference are incorrect. For example, Reference number 8 is probably from a web page, but the URL is not listed. I think the description "Organization WH" is also inappropriate. Please double check all the descriptions in the Reference list.

**Replay#5:** Thank you for your detailed remarks; please see the reference section for answers to all of your questions. Please see reference number 7 for more information.
